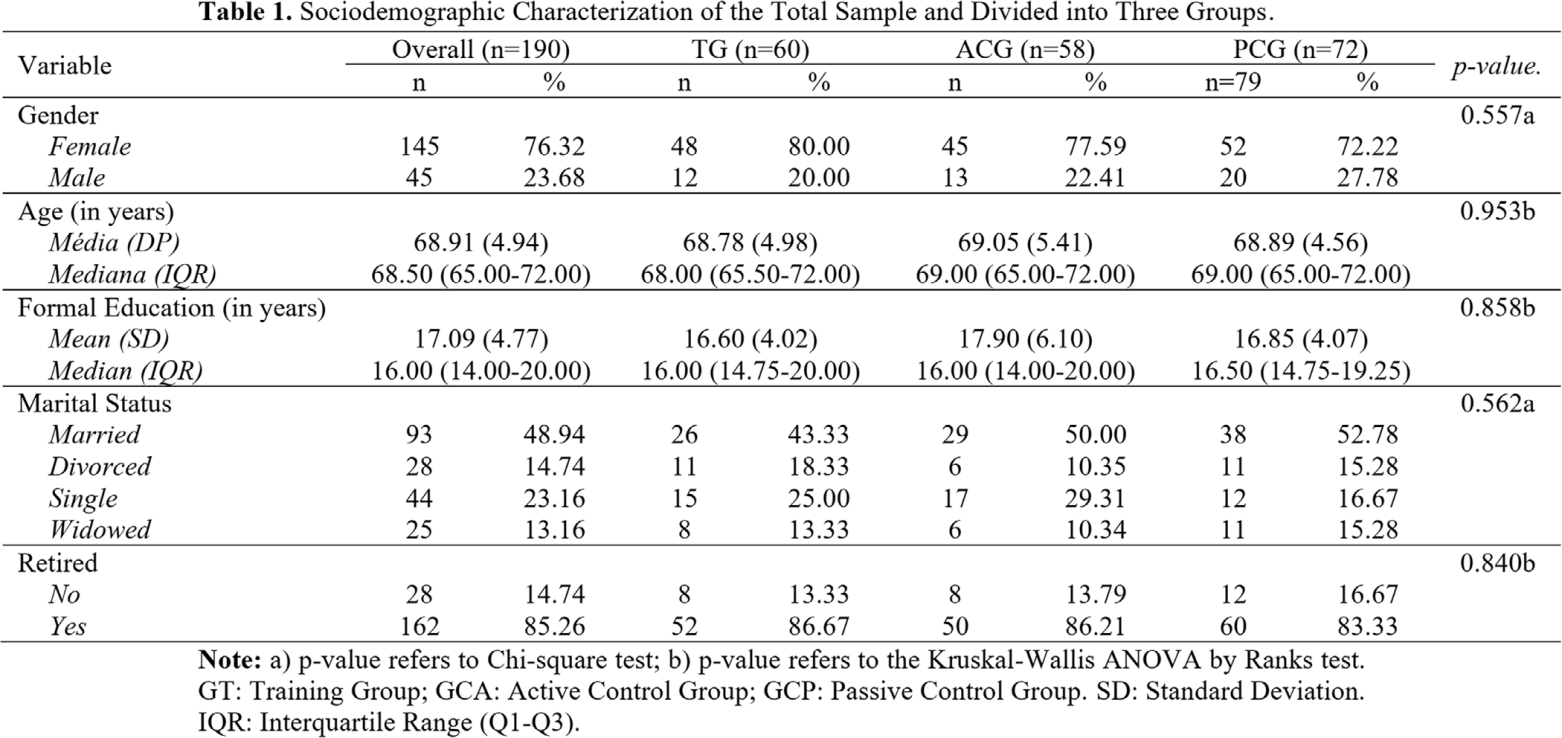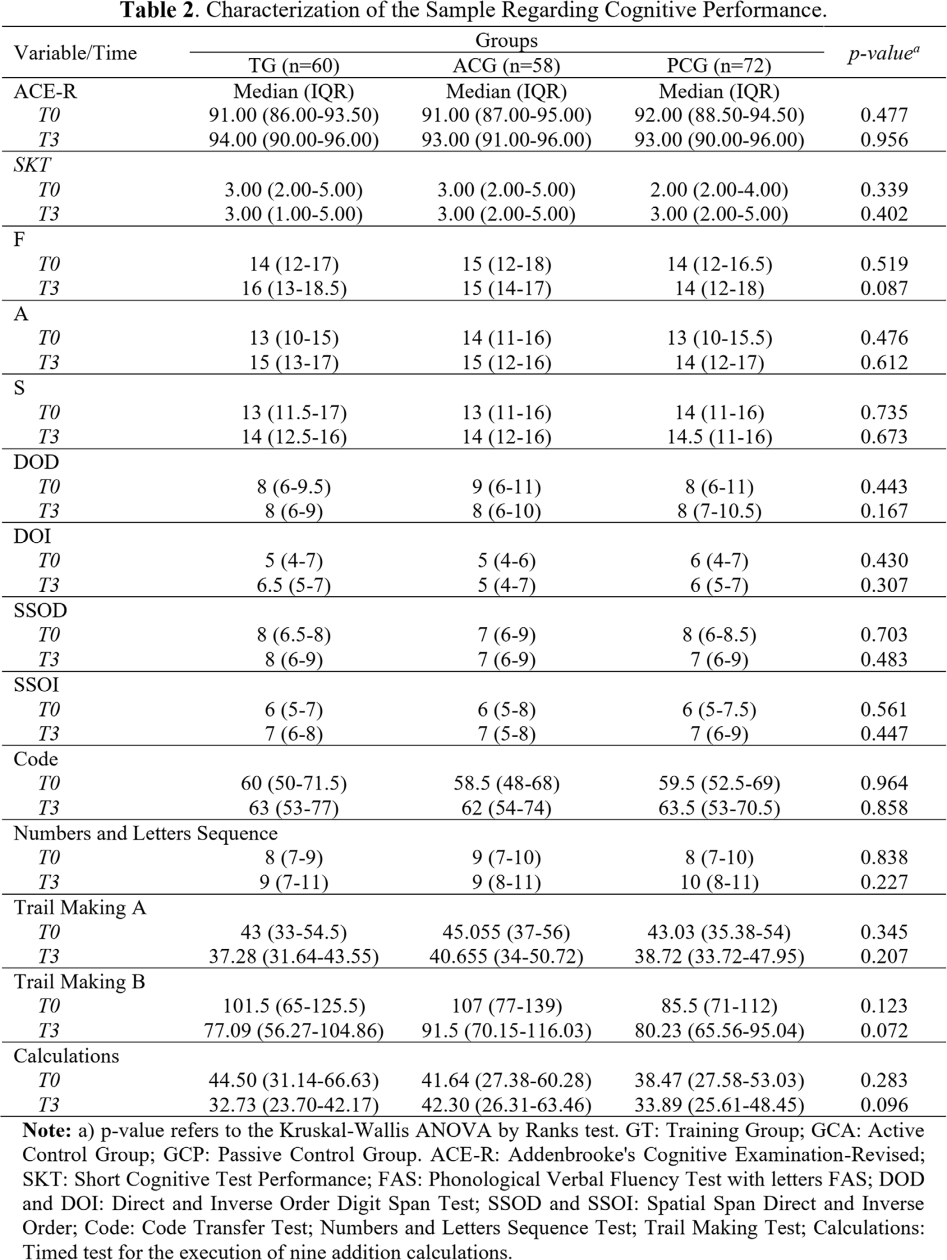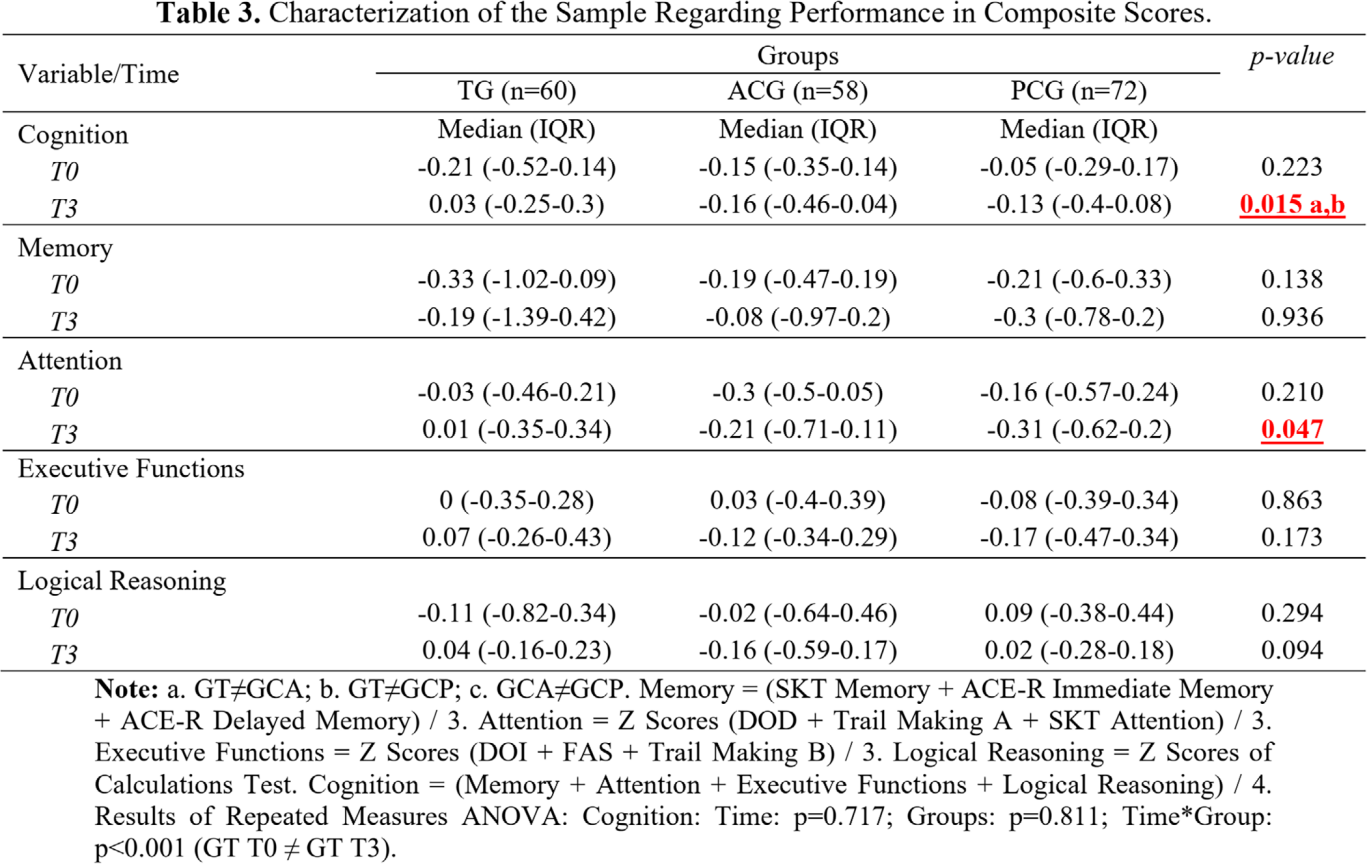# Cognitive performance assessment in healthy older adults: Results from an 18‐month multicomponent cognitive stimulation randomized clinical trial

**DOI:** 10.1002/alz.089969

**Published:** 2025-01-03

**Authors:** Thais Bento Lima Silva, Tiago Nascimento Ordonez, Gabriela dos Santos, Ana Paula Bagli Moreira, Laydiane Alves Costa, Bárbara Perpétuo, Patrícia Prata Lessa, Luiz Carlos Moraes, Neide Pereira Cardoso, Sonia Maria Dozzi Brucki, Monica Sanches Yassuda

**Affiliations:** ^1^ University of São Paulo, São Paulo, São Paulo Brazil; ^2^ Cognitive and Behavioral Neurology Unit (GNCC) of the University of São Paulo., São Paulo, São Paulo Brazil; ^3^ Universidade de São Paulo (USP), São Paulo, São Paulo Brazil; ^4^ USP, São Paulo, Brazil Brazil; ^5^ Universidade de São Paulo, São Paulo, São Paulo Brazil; ^6^ Supera Institute of Education, São José dos Campos, São Paulo Brazil; ^7^ Cognitive and Behavioral Neurology Unit, Medical School, University of São Paulo, São Paulo Brazil; ^8^ University of São Paulo, São Paulo Brazil; ^9^ Cognitive and Behavioural Neurology Unit ‐ University of São Paulo, São Paulo Brazil; ^10^ Hospital Santa Marcelina, Sao Paulo Brazil; ^11^ Medical School of University of São Paulo, São Paulo Brazil; ^12^ University of São Paulo Medical School, São Paulo Brazil; ^13^ Cognitive and Behavioral Neurology Unit ‐ University of São Paulo, São Paulo, Brazil, Sao Paulo Brazil; ^14^ University of São Paulo, SAO PAULO, SAO PAULO Brazil; ^15^ State University of Campinas UNICAMP, Campinas Brazil; ^16^ Universidade de São Paulo, São Paulo Brazil

## Abstract

**Background:**

This study investigated the effects of cognitive stimulation on older adults over 18 months through a randomized clinical trial with 190 participants divided into Training Group (TG), Active Control Group (ACG), and Passive Control Group (PCG). Initial sociodemographic characterization (Table 1) ensured homogeneity among the groups. The clinical trial design aimed to assess the long‐term impacts of multicompartment cognitive stimulation on the cognitive function of older adults in the TG.

**Method:**

Participants were randomly assigned to TG, ACG, or PCG, with an assessment of initial sociodemographic characteristics to ensure homogeneity. The analysis of cognitive performance (Table 2) and the development of the composite score (Table 3) were conducted. Using standardized z scores and specific formulas, the composite score incorporated assessments of memory, attention, executive functions, and logical reasoning, providing a comprehensive and balanced measure of cognitive function.

**Result:**

The evaluation of cognitive performance (Table 2) using individual instruments did not identify significant variations between the groups. However, composite scores (Table 3) indicated notable improvements in overall cognition for the TG compared to the other groups. Repeated measures ANOVA revealed significant differences in the composite cognition score for TG participants over time (T0 versus T3).

**Conclusion:**

The results highlight the effectiveness of long‐term cognitive stimulation, especially in the TG, suggesting measurable benefits in the cognition of healthy older adults. This study contributes to the development of a composite score for analyzing cognition in healthy older adults, utilizing nationally and internationally validated instruments. Additionally, it provides insights into the effects of long‐term multicompartment cognitive stimulation, guiding future interventions to promote cognitive health in older adults.